# Community engagement in early childhood development intervention trials: lessons learnt from a stepping stones program in rural India

**DOI:** 10.3389/fpubh.2025.1611971

**Published:** 2025-08-05

**Authors:** Zahiruddin Quazi Syed, Shital Telrandhe, Penny A. Holding, Swati Dudhkohale, Manoj Patil, Roshan Umate, Mahalaqua Nazli Khatib, Shilpa Gaidhane, Sonali G. Choudhari, Deepak Saxena, Abhay Gaidhane

**Affiliations:** ^1^Datta Meghe Institute of Higher Education and Research Deemed to be University, Department of Community Medicine, Jawaharlal Nehru Medical College, Wardha, Maharashtra, India; ^2^Datta Meghe Institute of Higher Education and Research Deemed to be University, Centre of Early Childhood Development, Datta Meghe Institute of Higher Education and Research, Wardha, Maharashtra, India; ^3^Datta Meghe Institute of Higher Education and Research Deemed to be University, Global International, Wardha, Maharashtra, India; ^4^Datta Meghe Institute of Higher Education and Research Deemed to be University, Stepping Stones Program, Wardha, Maharashtra, India; ^5^Datta Meghe Institute of Higher Education and Research Deemed to be University, i-Health Consortium, School of Epidemiology and Public Health, Jawaharlal Nehru Medical College, Wardha, Maharashtra, India; ^6^Datta Meghe Institute of Higher Education and Research Deemed to be University, Centre of Early Childhood Development – Stepping Stones Project, Department of Research and Development, Wardha, Maharashtra, India; ^7^Datta Meghe Institute of Higher Education and Research Deemed to be University, Global Evidence Synthesis Initiative, Division of Evidence Synthesis, Department of Physiology, Jawaharlal Nehru Medical College, Wardha, Maharashtra, India; ^8^Datta Meghe Institute of Higher Education and Research Deemed to be University, Department of Medicine, Department of Clinical Epidemiology, Jawaharlal Nehru Medical College, Wardha, Maharashtra, India; ^9^Datta Meghe Institute of Higher Education and Research Deemed to be University, Department of Community Medicine, School of Epidemiology and Public Health, Jawaharlal Nehru Medical College, Wardha, Maharashtra, India; ^10^Indian Institute of Public Health Gandhinagar, Department of Community Medicine, Gandhinagar, Gujarat, India; ^11^Datta Meghe Institute of Higher Education and Research Deemed to be University, Centre of One Health, Department of Community Medicine, School of Epidemiology and Public Health, Jawaharlal Nehru Medical College, Wardha, Maharashtra, India

**Keywords:** community engagement, early child development, community engagement board, rural India, stakeholder engagement

## Abstract

**Background:**

Delivering Early Childhood Development (ECD) interventions in low-resource settings requires context-sensitive adaptive strategies grounded in community realities. The Stepping Stones Program, implemented in rural central India, sought to improve parenting practices and child development outcomes through structured community engagement. This paper presents the approach, implementation, and program impact, offering insights into building scalable, community-owned models for ECD.

**Methods:**

This mixed-methods study was embedded within a cluster-randomized controlled trial conducted in 106 villages across two rural blocks in Maharashtra, India. A total of 656 pregnant women were enrolled and followed until their child reached 24 months. Intervention clusters received a responsive parenting and nutrition program integrated with existing Anganwadi services, while control clusters received standard government services. Community engagement was operationalized through participatory planning, the Photostory approach for initiating dialogue with the community, and the establishment of Curriculum Development Committees and a Community Engagement Board. A Monitoring, Evaluation, and Learning framework supported real-time adaptations. Process indicators were analyzed descriptively; child development outcomes were assessed at 24 months using mixed-effects regression models.

**Results:**

The program demonstrated strong community participation, with 85% of households engaging in at least one activity and 70% regularly attending sessions. Fathers’ participation in caregiving sessions increased from 4.3 to 33.65%. Balsakhis (local female volunteers) independently delivered over 80% of sessions. Integration with government systems was achieved through policy alignment and involvement of ICDS functionaries in program delivery. At 24 months, the mean difference of the development score in all domains between the intervention and control arm was statistically significant (*p* > 0.05). Children in the lowest wealth quintile showed the largest improvements in cognitive (effect size: 0.92, *p* < 0.001), motor (0.72), and language development (0.79). Children below the poverty line also showed significant gains across developmental domains.

**Conclusion:**

Structured, participatory community engagement was critical to the successful implementation and impact of the Stepping Stones Program. The program evolved from a top-down intervention to a community-driven platform for nurturing care. Findings support the utility of an adaptive engagement framework in building equitable, sustainable, and scalable ECD interventions in low-resource settings.

## Background

Implementing integrated community-based field intervention trials is a complex process, demanding careful planning and adaptable strategies to navigate the challenges of real-world settings. Delivering these multifaceted programs in Low and Middle-Income Countries (LMIC) and resource-limited settings demands comprehensive, multi-sectoral strategies, not only to ensure successful implementation but also for long-term sustainability, acknowledging the influence of the complex interaction of factors influencing program outcomes (1, 2). For meaningful Community engagement, the trial team needs to develop a collaborative partnership and work synergistically with the community members and stakeholders at all stages of the study for the community’s wellbeing (1).

Community engagement in research extends beyond passive involvement and requires commitment to shared decision-making, mutual respect, and a deep understanding of the community’s unique context, values, and priorities. This collaborative approach leverages community strengths and actively translates knowledge into actionable strategies for enhancing community health (1, 3).

Perceptions held by local communities and caregivers exert a substantial influence on the promotion of Early Childhood Development, underscoring the critical need to understand and integrate these perspectives into intervention strategies (1, 4). Understanding these perceptions can facilitate the adaptation of interventions to better align with local needs. Such engagement is critical for effectively scaling up ECD programs, ensuring that interventions are not only evidence-based but also contextually relevant and culturally appropriate (1, 4–6). Therefore, the field intervention trials aimed at promoting ECD must actively engage with diverse stakeholder groups, including caregivers, families, local communities, private preschools, and government ECD programs, to plan, implement, and evaluate program success (1–3).

This paper presents lessons learned from the community engagement process during the planning, implementation, and evaluation of an early childhood intervention in rural central India. The aim is to provide valuable insights for ECD program managers and service providers to enhance the effective delivery of ECD interventions at scale, particularly in remote and disadvantaged rural populations in India.

### Overview of the stepping stones program

In August 2014, we launched the Stepping Stones Program to promote Early Childhood Development (ECD) in rural and tribal communities in central India. The program aimed to empower parents and caregivers by strengthening their skills to create nurturing home environments during the first 1,000 days of a child’s life—a critical window for development and wellbeing.

The intervention was designed to be delivered through a structured approach, integrating Anganwadi centers and home visits facilitated by Anganwadi Workers (AWWs). As part of India’s Integrated Child Development Services (ICDS) scheme, Anganwadi centers provide non-formal education and nutrition to children under five. The program aimed to create an engaging and supportive learning environment through home visits, group sessions, and community meetings. AWWs played a pivotal role in bridging the gap between the intervention and the community, ensuring localized implementation (7, 8).

The program’s effectiveness was evaluated through a cluster-randomized trial (cRCT) with 656 participants. Clusters were randomly assigned to either the intervention or control group, allowing for a rigorous assessment of the program’s impact while accounting for intra-community variations. The goal was to generate evidence on the effectiveness of interventions during the crucial first 1,000 days of life. Detailed methodology and findings are available in the primary publication (8, 9).

### The settings and context

We implemented the *Stepping Stones* Program in underserved, rural, and socioeconomically disadvantaged areas in two blocks of the Wardha and Nagpur districts in Maharashtra, Central India. The Project site represents one of the region’s poorest populations, with an annual per capita income below the state average. Many individuals rely on daily wage labor, with only a few owning farmland. Household incomes, often dependent on forest and farm produce, have declined due to erratic monsoons and climate change. Traditional childcare practices are widespread and significantly influence health-seeking behavior. Access to health and social services remains a considerable challenge. Women in these areas generally have low educational attainment and limited knowledge, skills, and resources for responsive parenting. Social and economic distress further contributes to inadequate parenting, negatively impacting early childhood growth and development.

In India, Anganwadi centers provide ECD services under the Integrated Child Development Scheme (ICDS). Our intervention was integrated at the village level through these centers. Launched in the 1970s and subsequently scaled nationwide, ICDS is one of the world’s largest child development programs. Each Anganwadi center, catering to a population of approximately 1,000 people, is staffed by an Anganwadi Worker (AWW) and a helper. These workers deliver non-formal preschool education, nutritional supplementation, and anthropometric tracking for children under 6 years old, in addition to assisting health department staff in implementing national health programs, such as immunization and antenatal care. Evaluations of the ICDS program have highlighted the need to strengthen AWW capacity and enhance the quality of services, particularly in ECD (10, 11). Our program sought to address these gaps by equipping AWWs with the necessary skills and knowledge to improve early childhood outcomes in resource-limited settings.

## Methodology

### Study design

This study was embedded within a community-based Early Childhood Development (ECD) intervention trial known as the Stepping Stones Program, implemented in rural and peri-urban communities. The trial followed a mixed-methods implementation research design, combining both qualitative and quantitative approaches to evaluate and iteratively refine the intervention.

The stepping stones program’s impact was evaluated using a cluster-randomized design, with the Anganwadi Center (AWC) serving a population of 1,000 as the unit of randomization. We identified 50 clusters, each with at least 30 children under 60 months of age. Clusters were randomly allocated to the intervention and control arms (25 clusters in each arm). Caregivers from the intervention villages received the responsive parenting program over 9 months and the routine services delivered through AWC. In contrast, caregivers from the control villages received the standard services provided through Anganwadi centers (8).

### Randomization and allocation

The study was conducted in two adjacent administrative blocks—Seloo and Hingna—in central India, selected due to the research team’s established community engagement and logistical feasibility. To minimize contamination between intervention and control groups, the sub-center (serving 5–6 villages) was chosen as the unit of randomization. Using a stratified randomization approach, two PHCs were randomly selected from each block, yielding a total of 21 sub-centers (clusters) across 106 villages. Clusters were randomly assigned to intervention (11 sub-centers, 58 villages) and control arms (10 sub-centers, 48 villages) using an allocation sequence masked from the implementation team. Outcome assessors were blinded to intervention status, rotated across clusters to reduce familiarity bias, and trained extensively in tool administration. Refresher sessions and real-time monitoring were employed to ensure data quality and fidelity of outcome assessments.

### Study participants

Study participants were pregnant women in their first or second trimester, permanently residing in the selected study villages. Recruitment was conducted over a six-month period. Women were eligible if they met the gestational age criteria and provided written informed consent; those with high-risk pregnancies were excluded. All eligible participants were counselled regarding the purpose and procedures of the study, and informed consent was obtained from caregivers prior to enrollment. The enrolled women and their children were followed from pregnancy through the child’s second birthday.

### Ethics approval

Ethical approval for the Stepping Stones trial was obtained from the Institutional Ethics Committee of Datta Meghe Institute of Medical Sciences (Deemed to be University), vide reference number DMIMS(DU)/IEC/2014–15/1203, dated 31 March 2015. The trial was prospectively registered with the International Standard Randomized Controlled Trial Registry (ISRCTN87426020). Study participants and their families were counselled about the study objectives and procedures, and written informed consent was obtained prior to enrolment. All study activities were conducted in accordance with established ethical standards and regulatory guidelines.

### Intervention design

The intervention aimed to enhance nurturing care practices and child development outcomes through structured caregiver and community engagement. The Stepping Stones Program initially began with a top-down design, focusing primarily on caregiver education.

A formative phase, including Focus Group Discussions (FDGs), informed the design by contextualizing local parenting practices. The finalized intervention comprised eight structured group sessions, delivered with the support of Anganwadi Workers (AWWs), focusing on responsive parenting and nurturing care. To ensure feasibility and sustainability, implementation was integrated with existing government services, with nutrition supplementation provided through Anganwadi centers and home visits by trained community volunteers (Balsakhis) under AWW mentorship. The intervention targeted six domains essential to early childhood development: food and nutrition, shelter and care, health and safety, play and learning, psychosocial skills, and child protection, with additional components such as toy-making workshops and kitchen gardening to enhance caregiver engagement and child outcomes.

Interventions were delivered by Balsakhis, local women from the same village, typically married, literate, and available for 3 h daily. Balsakhi was trained and certified by the core Project team. The training emphasized a self-reflective, experiential learning approach and included role-plays, group discussions, and hands-on activities. Certification was based on observed performance during mock sessions and activity trials using standardized checklists. Intervention delivery involved both group sessions and home visits, with flexible scheduling tailored to local contexts and staff availability. Beneficiaries, including pregnant women and mothers of young children, were identified through baseline data, and session delivery was adapted to meet their needs effectively.

#### Adaptation based on initial community feedback

Following the initial implementation phase, the program encountered resistance from some community members, who expressed confidence in traditional child-rearing practices and questioned the relevance of external interventions. These early challenges highlighted the need for deeper engagement and listening. The intervention, Stepping Stones, was systematically co-developed by the trial team in collaboration with experts in early childhood development, public health, and government ECD stakeholders.

To foster contextual understanding and reflection, a “Photostory approach” (7) was used at baseline and during implementation. Community members, especially mothers, fathers, and caregivers, shared caregiving narratives and photographs, which were analysed to surface prevailing practices, beliefs, and opportunities for program alignment.

The “Curriculum Development Committee” was formed, consisting of community members, Anganwadi workers, local leaders, and preschool teachers. These committees guided the co-design of content and delivery strategies. The revised curriculum incorporated findings from Focus Group Discussions (FGDs) held with caregivers, front-line workers, and community influencers.

#### Community engagement board (CEB)

As part of our participatory implementation approach, a Community Engagement Board was constituted, comprising caregivers, Anganwadi workers, Balsakhis, Panchayat members, and frontline health staff. The CEB served as a platform for shared decision-making, addressing local challenges, and ensuring alignment between community needs and program priorities. Meeting quarterly, the Board reviewed progress, provided feedback on intervention strategies, and supported the adaptation and integration of activities into existing local systems. This structure fostered transparency, local accountability, and a sense of collective ownership, contributing to the sustainability of the ECD program.

Stakeholder mapping and policy alignment were conducted to ensure integration of the intervention within the existing ICDS and health systems. Balsakhis, local trained volunteers, were mobilized as key liaisons, bridging community realities and program implementation. These efforts were designed to enhance sustainability and community ownership.

#### Monitoring, evaluation, and learning (MEL)

An adaptive Monitoring, Evaluation, and Learning (MEL) framework supported real-time program learning and accountability. The framework drew on multiple data sources, including routine coverage and service delivery data, field notes and observational logs, caregiver feedback, structured debriefings, and community-generated photo documentation. Monthly Experience Sharing and Review Meetings (ESRMs) were held with stakeholders to review progress, identify gaps, and implement corrective actions. A Rapid-cycle Evaluation and Learning (REAL) model ensured dynamic course corrections based on data and community input (12).

The enhanced intervention framework reflected these adaptations and is detailed in Table 1.

**Table 1:** Enhanced intervention strategy of the stepping stones program developed through a collaborative community engagement process.

Implementation strategy	Objective/purpose	Approach/method
Home visits	To enhance parental knowledge and skills for responsive caregiving and creating a developmentally rich home environment for children aged 0–3 years.	Fortnightly visits conducted by trained community volunteers (*Balsakhis*) providing personalized guidance and support to caregivers. Sessions focused on responsive parenting, stimulation, and age-appropriate practices.
Enhanced ECD curriculum	To promote cognitive, emotional, and social development in children aged 3–6 years through structured, play-based learning.	Delivered by trained Anganwadi Workers (AWWs) with project staff support. The curriculum integrated interactive activities and locally relevant play materials. AWWs were provided with hands-on training, tools, and ongoing supervision.
Nutrition intervention: garden and demos	To improve caregiver knowledge and practice related to complementary feeding and nutrition.	Two-pronged approach: (i) *Nutrition Gardens* established at the household level with training to grow local food; (ii) *Nutrition Demonstration Centers*offered practical cooking sessions showcasing affordable, nutritious recipes using locally available ingredients.
Community workshops	To build caregiver capacity for low-cost play material creation and to foster peer learning.	Monthly workshops in each study village, led by *Balsakhis* and project staff. Caregivers were trained to make toys using locally available materials and encouraged to share their experiences, successes, and challenges.
Sensitization and group meetings	To raise awareness on early childhood development and nutrition and foster community ownership and participation.	Quarterly meetings at sub-health centers facilitated by project staff, AWWs, and community volunteers. These sessions included information dissemination, issue resolution, and collective reflection on program progress and local priorities.
Father and male caregiver engagement	To increase participation of fathers in caregiving and early stimulation.	Sessions specifically designed for fathers were conducted during community meetings and home visits, addressing their role in nurturing care and promoting joint responsibility in parenting.
Real-time feedback and review mechanisms	To ensure program adaptability and responsiveness to community needs.	Feedback loops were established through Experience Sharing and Review Meetings (ESRM), suggestion boxes, and informal debriefs. Input from caregivers and frontline workers informed ongoing refinement of content and delivery strategies.
Monitoring, learning, and adaptation (MEL)	To track program fidelity, responsiveness, and outcomes through a participatory, adaptive learning process.	A Rapid-cycle Evaluation and Learning (REAL) approach was used, incorporating community feedback, field observations, and quantitative data to enable timely course corrections. The MEL process was inclusive and transparent, involving all key stakeholders.

### Measures and assessments

At recruitment, the study team collected household sociodemographic data and antenatal characteristics of pregnant women using the Government of India National Family Health Survey (NFHS-5) tool (13).

Child development outcomes were evaluated using a standardized battery of assessment tools administered at 12 and 24 months of age. The selected tools have been previously validated and widely utilized in low-and middle-income country settings. Following comprehensive training and certification, trained field staff conducted the assessments. Among the tools used, the Developmental Milestones Checklist (DMC) is recognized for its reliability and sensitivity in assessing motor, language, and personal-social domains of development (14, 15).

The Profile of Socio-Emotional Development (PSED) was employed to assess children’s social and emotional development through direct observation and parental reports (16). The PSED tool was adapted to the local context, incorporating culturally and socially relevant items to enhance contextual validity. The quality of the home environment was assessed at baseline and end line using the adapted Home Scale Coding and Infant-Toddler Home Inventory (17–19). Mother–child interaction quality was evaluated using the Observation of Mother–Child Interaction (OMCI) tool (20), and parental behaviors, knowledge, and skills related to early childhood development were assessed using the Photostory approach and a structured parental quiz (7, 9). Anthropometric measurements were conducted by standardized World Health Organization (WHO) protocols, and all instruments were calibrated before use (21).

The study team adapted outcome assessment tools to the local context and translated them from English to Marathi (the local language), and back-translated them into English. The translated tools were also validated by language experts/teachers from the University. The data team developed an XLS file for all tools and then imported those files to Open Data Kit (ODK). Data collection tools were then imported into an Android Tablet-PC. The app had in-built quality checks that monitor score distributions and missing values/data. Using a tablet PC for data collection, we trained evaluators in the ODK process.

#### Assessment timeline

At recruitment, we captured the household information and mothers’ maternal characteristics using the DHS tool of the Government of India. At 12 months and 24 months, we assessed all child development outcomes (primary outcomes)—physical, cognitive, language, and socioemotional development. We also evaluated mother–child interaction and home environment at 12 and 24 months of intervention. Outcome assessors were blinded to the arm of the village. To ensure the quality and consistency of data, inter-rater reliability and intra-rater reliability testing were done. A team of enumerators collected the baseline information at recruitment from the intervention and the control arm. The trained and certified field officer administered all assessments. The assessors were blinded and independent of the implementing team.

### Analysis

#### Analysis of the process indicators

The process evaluation focused on assessing the fidelity, reach, quality, and adaptiveness of the Stepping Stones Program using data captured through a robust Monitoring, Evaluation, and Learning (MEL) framework. Quantitative indicators included participation rates, session coverage, fidelity of session delivery, quality of caregiver interactions, and male engagement. These were analyzed across time points to evaluate changes in implementation quality and coverage.

Monitoring data from Balsakhi field reports, session observation checklists, and attendance registers were aggregated quarterly and analyzed descriptively. Trends in participation, session quality (measured by duration and interactivity), and community contributions were tracked over the implementation period. Qualitative data from community feedback, experience-sharing meetings, and Photostory narratives were thematically analyzed to identify adaptation points and inform real-time improvements.

Key implementation indicators (e.g., percentage of sessions facilitated independently, percentage of interactive home visits, participation of fathers and other family members) were examined over six quarters to evaluate capacity building and community ownership. Integration with government systems was assessed through documentation of joint activities, participation of Anganwadi workers, and formal endorsement of program elements by local administrative bodies.

#### Impact evaluation

Conducted at the end of the intervention when participating children reached 24 months of age. The analysis employed mixed-effects linear regression models to estimate the intervention effect on child development outcomes, adjusting for relevant covariates including child age and sex, parental education and occupation, household wealth quintile, and family size. Random intercepts for clusters and assessors were included to account for intra-cluster correlation and measurement variability.

Outcomes were presented as adjusted mean differences between intervention and control arms, and standardized effect sizes (Cohen’s d) were calculated to facilitate interpretation of magnitude. Subgroup analyses were conducted to explore differential effects by socioeconomic status, particularly among children in the lowest wealth quintile and those living below the poverty line.

## Results

We present the results in two parts: (1) improvements in program quality and coverage, and (2) assessment of the intervention’s impact.

### Improvements in program quality and coverage

The Stepping Stones Program demonstrated notable improvements in both the quality and reach of its implementation using a robust Monitoring, Evaluation, and Learning (MEL) framework. The Community Engagement approach successfully operationalized a shift from a top-down intervention model to a participatory and contextually grounded approach. Curriculum Development Committees in all implementation clusters contributed to iterative design revisions. Key results included:

#### High levels of participation

Over 85% of eligible households engaged in at least one program activity, and 70% regularly attended sessions.

#### Increased father involvement

Photostory activities led to an increase in participation of fathers in caregiving dialogues (from 4.3% in the first quarter to 33.65% in the sixth quarter).

#### Improved monitoring and adaptive learning

The MEL system facilitated ongoing course corrections during the intervention. For instance, Child stimulation activities were modified after community feedback revealed low acceptability of certain materials. Gender-specific content was developed in response to caregiver and FGD suggestions on involving fathers and grandmothers. Balsakhi were trained to deliver intervention in a gender-sensitive manner.

#### Improved program coverage

We measured coverage by comparing the number of caregivers attending parenting sessions to our target. In the first quarter, less than one-third of the target group received services. By the sixth quarter, nearly 90% of eligible families had been reached.

#### Program quality

**Figure 1:**
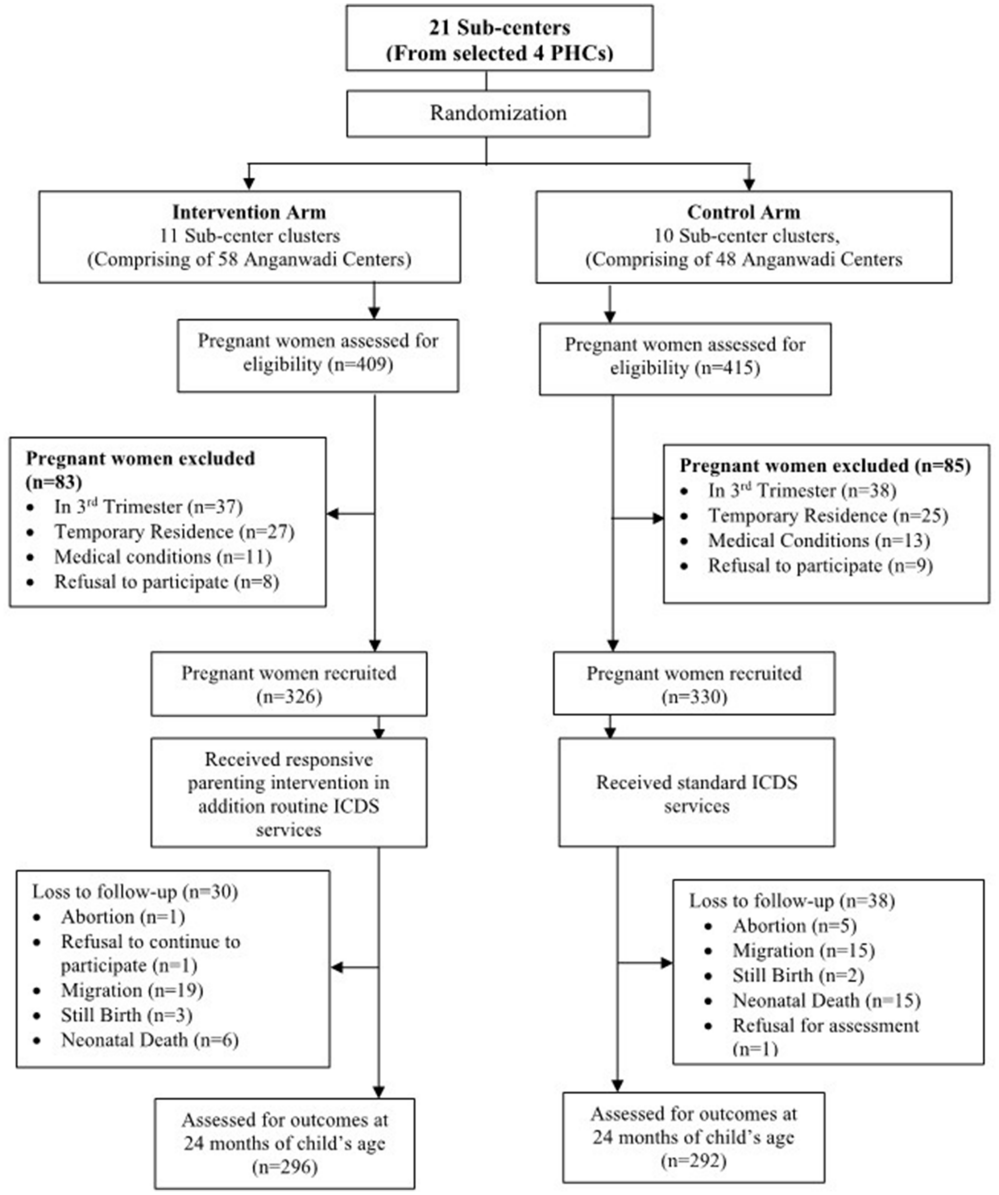
CONSORT flow diagram of the study-adapted from Gaidhane et al. (8).

**Figure 2:**
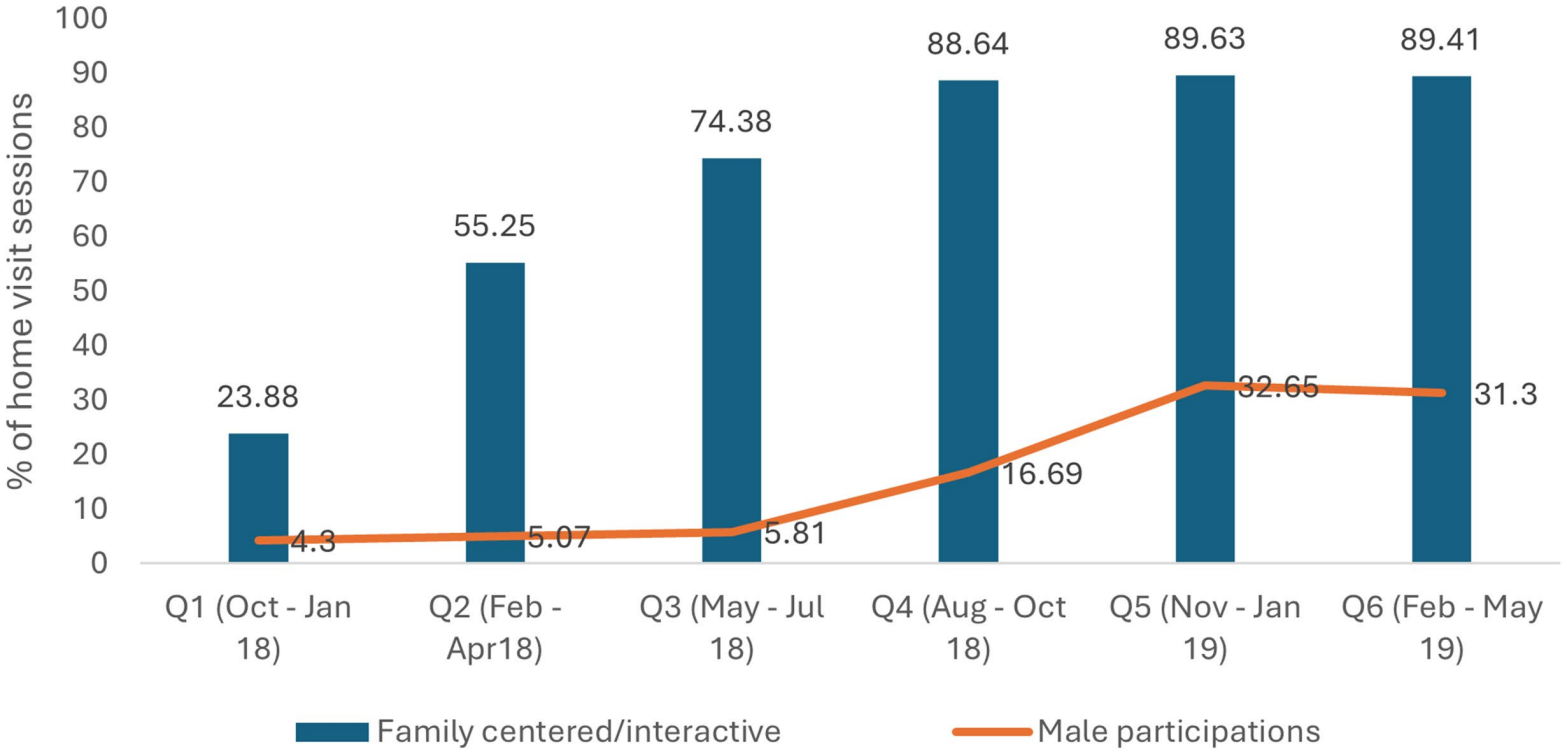
Quality (interactive and male participation) of home visit session delivered.

Balsakhis facilitated >80% of sessions independently as per the desired quality by the third month of implementation, reflecting capacity-building success. Initially, only 38% of visits lasted the intended 35–45 min, but by the sixth quarter, this rose to 89% (Figure 1). Additionally, the nature of interactions also shifted. Early sessions were largely directive and focused on information-sharing; by the sixth quarter, 90% of home visits were interactive and family-centered (Figure 2).

#### Integration with government system

Stakeholder mapping enabled strategic alignment with local ICDS, Health Department, and Panchayat leadership. As a result, government partners formally endorsed the adapted curriculum in two districts. ICDS functionaries provided supervision and infrastructure support in around 25% of the total sessions as planned. Anganwadi workers’ active participation in planning and review meetings increased from 22% at baseline to 68% post-intervention.

### Programs impact

We assessed the overall impact of the program when the child reached 24 months of age. Mixed-effects models were used to estimate the intervention effect, adjusting for key covariates including child age and sex, parental education and occupation, household wealth quintile, and family size. Clusters and assessors were included as random effects to account for clustering and measurement variability. Table 2 presents the result as a mean difference in the intervention in the control arm at 24 months. The mean difference was found to be statistically significant across multiple development domains, with the maximum mean difference seen for the cognitive development. Further details on the impact of the Stepping Stones Program on child development outcomes (standardized effect sizes - Cohen’s d) are presented in the primary results paper (8).

**Table 2:** Mean difference in the development outcome between the intervention and the control group at 24 months.

Development outcomes	Intervention	Control	Mean difference (95% CI)	*p*-value
Cognitive	70.18 (69.18 71.18)	67.65 (66.53 68.77)	2.53 (1.03 4.02)	0.001
Motor	53.36 (52.76 53.96)	51.97 (51.28 52.67)	1.38 (0.47 2.29)	0.002
Language	18.25 (17.70 18.81)	17.41 (16.79 18.03)	0.84 (0.01 1.67)	0.046
Socio-emotional development	21.47 (20.88 22.07)	20.51 (19.86 21.16)	0.96 (0.08 1.84)	0.031
Home scale score	36.58 (36.08 37.06)	35.61 (35.07 36.14)	0.96 (0.24 1.69)	0.008
Child score	12.3 (11.98 12.61)	11.64 (11.28 12.00)	0.65 (0.18 1.13)	0.006
Mother score	28.07 (27.69 28.45)	26.55 (26.13 26.98)	1.51 (0.94 2.08)	0.000
Mother child interaction	40.37 (39.75 40.99)	38.2 (37.50 38.89)	2.17 (1.24 3.10)	0.000

At 24 months, children from the lowest wealth quintile demonstrated the largest gains in cognitive (effect size: 0.92, *p* < 0.001), motor (0.72, *p* = 0.001), and language development (0.79, *p* < 0.001) compared to their more affluent counterparts. Similarly, children below the poverty line exhibited statistically significant improvements in cognitive (0.43, *p* < 0.001), language (0.25, *p* = 0.032), and PSED (0.36, *p* = 0.006) domains. Our community engagement strategy prioritized the inclusion of marginalized and socioeconomically disadvantaged populations from the outset (Table 3).

**Table 3:** Effect of intervention in various population subgroups at 24 months of age.

Variables	Cognitive		Motor		Lang		PSED	
	Effect size (95% CI)	*p-*value	Effect size (95% CI)	*p-*value	Effect size (95% CI)	*p-*value	Effect size (95% CI)	*p-*value
Wealth quintiles								
Lowest	0.92 (0.53 1.30)	0.000	0.72 (0.29 1.14)	0.001	0.79 (0.43 1.16)	0.000	0.18 (−0.28 0.64)	0.442
Lower–Middle	0.22 (−0.14 0.60)	0.239	0.13 (−0.27 0.52)	0.527	0.12 (−0.23 0.47)	0.491	0.28 (−0.08 0.64)	0.134
Middle	0.31 (−0.01 0.63)	0.058	0.22 (−0.12 0.55)	0.209	0.18 (−0.17 0.54)	0.317	0.36 (0.03 0.69)	0.029
Higher–Middle	minus 0.08 (−0.44 0.27)	0.648	0.16 (−0.28 0.60)	0.479	minus 0.03 (−0.4 0.33)	0.859	minus 0.01 (−0.38 0.34)	0.928
Highest	0.29 (−0.10 0.70)	0.145	0.14 (−0.15 0.43)	0.344	0.07 (−0.30 0.46)	0.692	0.21 (−0.15 0.56)	0.257
Sex								
Male	0.32 (0.11 0.54)	0.003	0.26 (0.01 0.51)	0.039	0.22 (0.01 0.44)	0.044	0.22 (−0.02 0.46)	0.074
Female	0.35 (0.08 0.62)	0.010	0.30 (0.07 0.53)	0.01	0.21 (−0.08 0.51)	0.158	0.27 (0.03 0.52)	0.024
Below poverty line (BPL)								
Yes	0.43 (0.19 0.67)	0.000	0.19 (−0.03 0.42)	0.090	0.25 (0.02 0.49)	0.032	0.36 (0.10 0.62)	0.006
No	0.21 (−0.03 0.46)	0.096	0.33 (0.10 0.56)	0.004	0.17 (−0.08 0.43)	0.181	0.06 (−0.19 0.32)	0.625

## Discussion

The implementation of the Stepping Stones program highlights how initial challenges, when addressed through critical reflection and active community engagement, can serve as catalysts for meaningful improvements in intervention design and delivery. The impact evaluation of the Stepping Stones program suggested that the program contributed to mild to moderate enhancements across key domains of ECD. The most substantial impact was observed in mother–child interactions (effect size: 0.4), indicating a significant improvement in responsive caregiving practices, which are well-established determinants of optimal early development.

Importantly, subgroup analyses demonstrated that the greatest developmental gains were among children from the most socioeconomically disadvantaged backgrounds. At 24 months, children in the lowest wealth quintile exhibited improvements in cognitive, motor, and language development. Likewise, children living below the poverty line showed significant progress in cognitive, language, and socioemotional domains. These findings highlight the program’s potential to advance equity by addressing early developmental disparities through targeted and inclusive approaches.

### Pathways to impact: from top-down design to community-driven intervention

The Stepping Stones Program evolved from a conventional top-down initiative into a co-created, community-driven model built on trust, cultural relevance, and shared ownership. The enhanced Stepping Stones Program introduced targeted strategies to comprehensively address ECD within the community. These efforts aimed to promote holistic child wellbeing while equipping parents with the knowledge and skills necessary to support their children’s development. Through a multifaceted and inclusive approach, the intervention fostered sustainable impacts by actively engaging both caregivers and the wider community. Below, we outlined the key strategies and processes that shaped this transition and contributed to the program’s effectiveness and community acceptance.

### Navigating initial challenges and building community trust

In the early phase of implementation, community members expressed resistance, questioning the relevance of external inputs on child-rearing. This feedback prompted critical reflection and led to a fundamental shift in the program’s engagement strategy. Community participation was no longer treated as a one-time, introductory activity but redefined as a continuous, responsive process. The team actively engaged caregivers, Anganwadi workers, ICDS staff, and local leaders through regular meetings, informal dialogues, and participatory tools such as Photostory sessions. These efforts fostered two-way communication and enabled real-time adaptation based on community input. Community engagement thus became a central operational pillar—integrated into all stages of the intervention—and played a key role in establishing early childhood development (ECD) as a shared local priority rather than an externally driven agenda.

#### Photostory approach: a participatory tool for dialogue and reflection

One of the program’s early turning points was the adoption of a Photostory Approach, a participatory visual-narrative method designed to stimulate structured community dialogue on ECD (7). Through photographs and personal stories shared by caregivers, including fathers, whose perspectives are often underrepresented, the program team gained insights into caregiving norms, cultural values, and gender dynamics. These narratives informed intervention design, helped document progress, and were showcased in community forums to celebrate achievements and reinforce ownership.

#### Co-creation through participatory planning

The program emphasized co-design through the establishment of a Curriculum Development Committee, which included community representatives, preschool educators, and front-line service providers from the health and women and child development departments. Content and delivery strategies were informed by extensive stakeholder consultations, including focus group discussions with Anganwadi workers, parents, and local leaders. This participatory approach ensured that the curriculum aligned with local needs, caregiving practices, and cultural values. Iterative piloting and refinement further enhanced the program’s acceptability, cultural congruence, and long-term sustainability.

#### Leveraging front-line providers and community volunteers as community connectors

Local community volunteers in the Stepping Stones Project, known as Balsakhis, played a pivotal role as intermediaries between the program team and community members. These front-line providers were trained in community engagement, supervision, and monitoring, enabling them to facilitate two-way communication and build trust. Their involvement ensured that program adaptations remained responsive to the evolving needs and perspectives of both service providers and beneficiaries.

#### Fostering a community-centric and equitable ECD ecosystem

A key innovation of the Stepping Stones Program was its transition from a mother-or caregiver-focused model to a broader, community-centric approach. Through participatory processes, the program identified and engaged a wide network of direct and indirect stakeholders, including caregivers, fathers, Anganwadi workers, ICDS staff, local leaders, and community influencers. This inclusive strategy not only expanded the scope of engagement but also strengthened collective responsibility for nurturing care and early childhood development (ECD).

To support this shift, a “Community Engagement Board” was established, comprising community representatives, service providers, and project staff. The board served as a platform for ongoing dialogue, co-design, and joint decision-making. It played a crucial role in aligning program content with cultural values, addressing emerging challenges, and enhancing transparency. This structure helped build local ownership and sustained participation, particularly among socioeconomically disadvantaged groups.

The Stepping Stones program aimed to be strategically aligned with government systems, and this was facilitated through the mapping of the policies that support and promote ECD and stakeholder engagement. As a result, the intervention fostered improvements across multiple levels: families reported positive developmental changes in children; frontline workers experienced stronger community collaboration; and government partners recognized alignment with policy goals. These efforts collectively created an enabling ecosystem—grounded in trust, shared ownership, and social cohesion—that contributed to the program’s scalability, sustainability, and equity-enhancing potential.

#### Monitoring, learning, and adaptive implementation

A distinguishing feature of the program was its cyclical Monitoring, Evaluation, and Learning (MEL) framework, designed to support real-time adaptation and community-centered data use (22, 23). Drawing on field notes, visual documentation, and caregiver feedback, the MEL system functioned as a responsive learning loop. It helped to (i) manage complexity by tracking diverse components, (ii) adapt strategies to local sociocultural and economic contexts, (iii) drive decisions through collaborative analysis of coverage data and field insights, and (iv) enhance reach and delivery through timely course corrections. Embedding community voices in MEL processes promoted ownership, relevance, and scalability of the intervention.

The Stepping Stones program promoted transparency through “Community Data Sharing.” The program also implemented a “Rapid-cycle Evaluation and Learning (REAL)” approach to enhance transparency, mutual accountability, and community participation (12). Monthly Experience Sharing and Review Meetings facilitated joint interpretation of data and timely action. The program strengthened trust and promoted local ownership by clearly communicating how data would be used and involving community members in sharing the findings. Community representatives actively participated in presenting results through co-authored reports and presentations, which supported mutual learning and increased the credibility of the program within the community.

#### Upholding ethical and inclusive practices

Ethical integrity was central to program design and implementation. The team adopted a flexible ethical framework grounded in respect, equity, and transparency, which honored the values and diversity of the communities involved. This inclusive approach helped ensure that the intervention remained both technically sound and socially acceptable.

#### Challenges in implementation and lessons learned

Despite notable successes, the program faced several implementation challenges. These included navigating complex sociopolitical realities, such as caste, racial, and economic disparities, and addressing intra-community divisions that necessitated the adaptation of intervention across varied sociocultural contexts. Building trust in such heterogeneous settings required responsiveness and sustained effort. Ensuring long-term sustainability also posed a significant challenge, particularly in maintaining quality across diverse geographies. Regular training, mentorship, and supervision were essential for maintaining the motivation and performance of front-line workers, particularly Balsakhis. A structured support system and context-specific adaptations were crucial for achieving consistent implementation and community ownership across regions.

### A structured framework for community engagement in field-based ECD trials

To guide and assess community participation in our early childhood development (ECD) field intervention trial, we adopted a structured Community Engagement (CE) framework comprising five interrelated components: Intent, Awareness, Efforts, Service Responsiveness, and Consequences. This framework was adapted from the Measurement of for Change (M4C) approach and operationalized to support sustained, meaningful engagement across all phases of the intervention (23).

1. *Intent*: the foundational intent of community engagement was to enhance the acceptance, reach, and quality of the intervention by aligning it with the sociocultural context and aspirations of the local population. Early engagement with caregivers, families, and community leaders allowed for a deeper understanding of community priorities regarding child-rearing, aspirations for children’s development, and perceived barriers to nurturing care. These insights informed the initial design and adaptation of intervention components.
2. *Awareness*: awareness-building focused on creating a shared understanding of the intervention’s goals, rationale, and potential benefits. This was achieved through community meetings, participatory orientation sessions, and the dissemination of culturally appropriate communication materials. Emphasis was placed on the importance of responsive caregiving and the critical window of the first 1,000 days, helping to create a common knowledge base and increase motivation to engage.
3. *Efforts*: community engagement was intentionally integrated into every stage of the intervention cycle, from planning to monitoring and evaluation. Structured efforts included: community dialogues to inform program design and identify contextual needs; involvement of local volunteers (e.g., Balsakhis) in service delivery and facilitation of parent sessions; and inclusion of community voices in interpreting data and contextualizing outcomes. This ongoing participation helped ensure that the intervention remained relevant, adaptive, and grounded in the lived experiences of beneficiaries.
4. *Service responsiveness*: responsiveness emphasized the ethical obligation of implementers to remain transparent, accountable, and adaptive. Feedback mechanisms, such as suggestion boxes, routine review meetings, and facilitated discussions, enabled caregivers and local stakeholders to share concerns, experiences, and suggestions. These insights directly informed iterative refinements in service delivery, ensuring that the program remained responsive to evolving community needs.
5. *Consequences*: the cumulative impact of this structured engagement approach was reflected in the tangible outcomes observed at both individual and community levels. Enhanced trust and shared ownership contributed to improved enrolment rates, greater consistency in participation, and stronger perceived value of the program. These factors likely contributed to the improved developmental outcomes observed, especially among socioeconomically disadvantaged families.

Table 4 presents the detailed application of this framework in the context of the Stepping Stones Program.

**Table 4:** Detailed application of the community engagement framework in the context of the stepping stones program.

Component	Definition	Illustrative examples from the stepping stones program
Intent	The foundational purpose of engaging the community—to enhance the intervention’s relevance, acceptance, reach, and quality by aligning it with local needs and aspirations.	Early consultations with parents, caregivers, and local leaders to identify community priorities and tailor the intervention content accordingly. This helped shape culturally appropriate strategies.
Awareness	The process of informing and educating the community about the intervention’s goals, evidence base, and anticipated benefits, particularly emphasizing early development.	Conducted participatory workshops, community meetings, and distributed IEC materials highlighting the importance of nurturing care and the first 1,000 days in child development.
Efforts	The deliberate and sustained actions taken to involve the community across all phases—planning, implementation, monitoring, and evaluation.	Engaged *Balsakhis* as community volunteers for delivering sessions; conducted community dialogues to co-design the intervention; involved community members in interpreting Photostory data and monitoring outcomes.
Service Responsiveness	The accountability of implementers to adapt and improve services based on community feedback, ensuring quality and responsiveness to evolving needs.	Institutionalized feedback mechanisms (e.g., monthly experience sharing and review meetings); used caregiver suggestions to refine session delivery and content; ensured transparency on intervention progress.
Consequences	The tangible and intangible outcomes resulting from meaningful engagement—such as increased trust, ownership, participation, and improved developmental indicators.	Observed increased enrolment and retention among marginalized groups; improved caregiver-child interactions; strengthened community ownership, contributing to more equitable ECD outcomes.

## Conclusion

The evolution of the Stepping Stones Program demonstrates that structured, participatory community engagement can serve as a powerful mechanism for enhancing the relevance, effectiveness, and equity of early childhood development (ECD) interventions, particularly in resource-constrained settings. By systematically embedding a five-component engagement framework, the program moved from initial resistance to deep community ownership. This shift required inclusivity, adaptability, and sustained dialogue, ultimately transforming the intervention into a community-driven platform for nurturing care. The framework offers a replicable model for future ECD initiatives seeking to build authentic partnerships with communities and to design interventions that are not only scalable and sustainable but also grounded in the lived experiences of those they aim to serve.

## Data Availability

The raw data supporting the conclusions of this article will be made available upon reasonable request. Requests to access these datasets should be directed to ST, shital.telrandhe88@gmail.com.
